# *Mycobacterium tuberculosis* Rv2145c Promotes Intracellular Survival by STAT3 and IL-10 Receptor Signaling

**DOI:** 10.3389/fimmu.2021.666293

**Published:** 2021-05-04

**Authors:** Hye-Soo Park, Yong Woo Back, In-Taek Jang, Kang-In Lee, Yeo-Jin Son, Han-Gyu Choi, Thi Binh Dang, Hwa-Jung Kim

**Affiliations:** Department of Microbiology and Medical Science, and Translational Immunology Institute, College of Medicine, Chungnam National University, Daejeon, South Korea

**Keywords:** *Mycobacterium tuberculosis*, Rv2145c, IL-10, STAT3, pathogenic role

## Abstract

Although *Mycobacterium tuberculosis* (Mtb) is an intracellular pathogen in phagocytic cells, the factors and mechanisms by which they invade and persist in host cells are still not well understood. Characterization of the bacterial proteins modulating macrophage function is essential for understanding tuberculosis pathogenesis and bacterial virulence. Here we investigated the pathogenic role of the Rv2145c protein in stimulating IL-10 production. We first found that recombinant Rv2145c stimulated bone marrow-derived macrophages (BMDMs) to secrete IL-10, IL-6 and TNF-α but not IL-12p70 and to increase the expression of surface molecules through the MAPK, NF-κB, and TLR4 pathways and enhanced STAT3 activation and the expression of IL-10 receptor in Mtb-infected BMDMs. Rv2145c significantly enhanced intracellular Mtb growth in BMDMs compared with that in untreated cells, which was abrogated by STAT3 inhibition and IL-10 receptor (IL-10R) blockade. Expression of Rv2145c in *Mycobacterium smegmatis* (*M. smegmatis*) led to STAT3-dependent IL-10 production and enhancement of intracellular growth in BMDMs. Furthermore, the clearance of Rv2145c-expressing *M. smegmatis* in the lungs and spleens of mice was delayed, and these effects were abrogated by administration of anti-IL-10R antibodies. Finally, all mice infected with Rv2145c-expressing *M. smegmatis* died, but those infected with the vector control strain did not. Our data suggest that Rv2145c plays a role in creating a favorable environment for bacterial survival by modulating host signals.

## Introduction

*Mycobacterium tuberculosis* (Mtb) is the cause of tuberculosis (TB) and threatens global health, with 10 million ill people (1). The incomplete efficacy of the BCG vaccine and the appearance of drug resistant strains are the main hurdles for TB control. The identification and characterization of Mtb components related to pathogenesis or virulence are required to develop a new vaccine and drugs for TB.

Mtb, an airborne pathogen, is primarily predated by alveolar macrophages and is then cleared by the host immune system or manipulates immune responses to avoid death and to create an environment favorable for replication; therefore, it can survive and replicate in a hostile environment (2, 3). Mtb has evolved multiple strategies to avoid an efficient host eradication mechanism. The primary survival mechanism is that mycobacteria inhibit phagolysosome fusion, and several mycobacterial factors, such as PtpA (4), ManLAM (5), and SapM (6), are involved in this inhibition. As another mechanism, virulent Mtb modulates host macrophage death, inhibiting apoptosis and triggering necrosis. The Mtb *nuoG* gene (7), SecA2 (8) and pknE (9) proteins are implicated in the inhibition of host cell apoptosis. Other mycobacterial lipids and proteins are responsible for mycobacterial survival and persistence (10), such as avoidance of antigen presentation (11) and modulation of the host cell signal network (12).

Hosts induce diverse innate and adaptive immune responses during Mtb infection to contain bacteria and produce pro- and anti-inflammatory cytokines against mycobacterial infection. IFN-γ plays a critical role in activating macrophages to kill bacteria. In contrast, IL-10 is a potent immunosuppressive cytokine that affects the bactericidal capacity of macrophages. Blocking the action of IL-10 during chronic Mtb infection in mice stabilizes bacterial growth and improves mouse survival (13). The inhibition of STAT3, which is activated by IL-10, is detrimental for Mtb intracellular replication (14). Recently, peptide inhibitors targeting the IL-10-STAT3 pathway have been demonstrated as host-directed therapies (15). Therefore, it is well established that IL-10 represents one such regulatory mechanism that Mtb could exploit to establish a chronic infection. The production of IL-10 in Mtb-infected macrophages is primarily an evasion mechanism against antimycobacterial reactions (16). One major mechanism of Mtb killing is mediated by IFN-γ activation of macrophages, but it can be inhibited by IL-10, and by blocking phagosome maturation through STAT3 signaling, which promotes Mtb survival and growth (17). However, little is known about mycobacterial components that directly stimulate IL-10 production from immune cells.

An increase in IL-10 expression in the lungs of chronically Mtb-infected mice has been demonstrated (15, 18). Mycobacterial PE (Pro-Glu) and PPE (Pro-Pro-Glu) proteins, which play important roles in pathogenesis, stimulate macrophages or dendritic cells to produce pro- and anti-inflammatory responses. Some proteins, such as PPE26 and PPE57, induce macrophage activation *via* TLR2 and promote pro-inflammatory cytokine production in macrophages (19, 20). PPE34 (Rv1917c) induces the maturation of dendritic cells and drives Th2 immune responses (21). In addition, PPE18 stimulates macrophages *via* TLR2 to secrete IL-10 (22). Although a few mycobacterial proteins inducing IL-10 production in immune cells have been reported, little is known about the detailed mechanism leading to bacterial survival by Mtb proteins that strongly stimulate IL-10 production.

We previously identified *Mycobacterium avium* subsp. *paratuberculosis* MAP1889c, which induces maturation of dendritic cells accompanied by elevated IL-10 production and Th2 responses (23). To investigate the pathogenic role of the protein in stimulating IL-10 production, we identified Mtb Rv2145c (Wag31) with sequence homology to MAP1889c and tested its direct effect on intracellular Mtb survival. In the present study, we found that recombinant Rv2145c stimulated macrophages to secrete higher IL-10 production and induced an increase in Mtb growth in macrophages. Signaling through STAT3 and the IL-10 receptor was required for Rv2145c-mediated Mtb growth enhancement. Furthermore, Rv2145c expression induced an increase in *Mycobacterium smegmatis* (*M. smegmatis*) growth *in vitro* and *in vivo*. These data provide possible evidence that Rv2145c may be a virulence factor of Mtb.

## Materials and Methods

### Ethics Statement

All animal experiments for this study followed the Korean Food and Drug Administration (KFDA) animal care and use guidelines. Animal experiments were performed according to procedures approved by the Institutional Animal Care and Use Committee of Chungnam National University, Daejeon, South Korea (permit number: CNU-01043).

### Mice and Cells

C57BL/6 (H-2Kb and I-Ab), C57BL/6J TLR2 knockout (TLR2^-/-^; B6.129-Tlr2tm1Kir/J), C57BL/10 TLR4 knockout (TLR4^-/-^; C57BL/10ScNJ), and BALB/c mice were purchased from the Jackson Laboratory (Bar Harbor, ME, USA). All mice were maintained under specific pathogen-free conditions in the Medical Research Center of Chungnam National University.

Bone marrow-derived macrophages (BMDMs) were cultured in Dulbecco’s modified Eagle’s medium (Welgene Co., Daegu, Korea) supplemented with 10% fetal bovine serum (FBS) (Welgene), 100 unit/ml penicillin/100 μg/ml streptomycin (Welgene) and 50 ng/ml mouse macrophage colony stimulating factor (M-CSF) (R&D Systems, Minneapolis, MN, USA) at 37°C and 5% CO_2_ for 6 days.

### Construction of Recombinant Proteins and *Mycobacterium smegmatis* (*M. smegmatis*) Strains

Transformation with recombinant proteins and recombinant Rv2145c-expressing *M. smegmatis* strains was performed as described previously (24). Fragments of Rv2145c, Ag85B, Rv2145c D1 and Rv2145c D2 were amplified from Mtb H37Rv ATCC 27294 genomic DNA by PCR using the appropriate primers (**Table S1**). The recombinant plasmids containing *Rv2145c*, *Ag85B*, *Rv2145c D1* and *Rv2145c D2* were transformed into *Escherichia coli* (*E. coli*) BL21 cells by heat shock for 1 min at 42°C.

*M. smegmatis* strain mc^2^155, grown in 7H9 medium supplemented with 10% oleic acid albumin dextrose catalase (OADC; BD Biosciences, San Jose, CA, USA) was harvested, washed with cold 10% glycerol and resuspended in the same buffer. The plasmids (pVV16 and pVV16-Rv2145c) were electroporated into the bacteria according to the standard procedure of the Gene Pulser apparatus (Bio-Rad, Hercules, CA, USA). The recombinant *M. smegmatis* strains were selected on 7H10 agar plates containing 50 μg/ml kanamycin (Sigma-Aldrich, St. Louis, MO, USA, 60615). Plates were incubated at 37°C for 3–4 days to obtain the recombinant strains.

### Bacterial Strains, Culture and Infection

Mtb H37Rv (ATCC 27294, Mtb) and *M. smegmatis* strain mc^2^155, obtained from American Type Culture Collection (ATCC, Manassas, VA), were grown in 7H9 medium supplemented with 0.5% glycerol and 10% OADC at 37°C. Kanamycin (50 μg/ml) was added to culture the recombinant *M. smegmatis* strains. Aliquoted bacteria stored at -80°C were thawed and plated onto 7H10 agar plates with or without 50 μg/ml kanamycin to enumerate the colony-forming units (CFUs).

For *in vitro* infections with Mtb, BMDMs were infected with Mtb (multiplicity of infection, MOI = 1) for 4 h were treated with gentamicin (200 μg/ml; Sigma) for 2 h kill noningested Mtb, washed with medium, and then incubated with antibiotic-free complete medium containing Rv2145c for the indicated additional time periods. To measure the number of bacteria, the cell lysates were plate onto 7H10 agar plates. For *M. smegmatis*, BMDMs were infected at an MOI of 10, treated with gentamicin (200 μg/ml) for 2 h to remove any remaining extracellular bacteria and then incubated in medium with 10% FBS containing gentamicin (20 μg/ml) for the indicated additional time period. The bacterial counts were performed using 7H10 agar plates containing kanamycin (50 μg/ml).

For *in vivo* infection with *M. smegmatis*, C57BL/6 mice were infected *via* the tail vein with the bacteria (using 1 × 10^6^ CFU/mouse) for 24, 72 or 168 h. To measure the bacterial burden in mice, the lung and spleen were homogenized in 1 ml PBS, and serial dilutions of the homogenates were plated on 7H10 agar plates containing kanamycin (50 μg/ml). CFUs were determined after incubation at 37°C.

### Antibodies and Reagents

Endotoxin removal resin (END-X B15) and an endotoxin filter (END-X) were purchased from the Associates of Cape Cod (East Falmouth, MA, USA). LPS from *Escherichia coli* O111:B4 (cat code. tlrl-eblps) and palmitory-3-Cys-Ser-(Lys)4 (Pam3CSK4, cat code. tlrl-pms) were purchased from InvivoGen (San Diego, CA, USA). A fluorescein isothiocyanate (FITC)-Annexin V Apoptosis Detection Kit (cat. 556547) and DAPI (cat. D3571) were purchased from BD Biosciences and Molecular Probes (Eugene, OR, USA), respectively. Specific inhibitors of STAT3 (S31-201, cat. 573102), ERK1/2 (U0126, cat. 662005), p38 (SB203580, cat. 559389), JNK (SP600125, cat. 420119) and NF-κB (BAY 11-7082, cat. 196870) were purchased from Calbiochem. Silencer negative control siRNA (AM4611), silencer predesigned siRNA STAT3 (cat. AM16708) and TurboFect transfection reagent were purchased from Thermo Fisher Scientific. Detailed information on the antibodies is listed in **Table S2**.

### Annexin V and PI Staining

BMDMs treated with Rv2145c or LPS (100 ng/ml) for 24 h were stained with Annexin V and PI according to manufacturer’s instruction and analyzed using a FACSCanto flow cytometer (BD Biosciences). FlowJo data analysis software (BD Biosciences) was used data processing.

### Sandwich ELISA Analysis

The cytokine levels were determined from the culture supernatants of BMDMs stimulated with various stimuli, including Rv2145c, or infected with mycobacteria with a Vmax kinetic microplate reader (Molecular Devices Co., Sunnyvale, CA, USA) according to the manufacturer’s instructions (eBioscience; San Diego, CA, USA).

### Flow Cytometry Analysis

After the appropriate stimulation or infection, the cells were harvested and preincubated with 0.5% BSA in PBS for 30 min. The cells were stained with fluorescence-conjugated antibodies for 30 min at room temperature. The intensity of expression of surface molecules and cytokine receptors was analyzed by flow cytometry (FACSCanto), and the data were processed using FlowJo data analysis software (BD Biosciences).

### Confirmation of LPS Decontamination of Recombinant Rv2145c

For heat-denaturation, LPS or Rv2145c were incubated at 100°C for 1 h. For digestion of proteinase K (PK), LPS or Rv2145c were incubated along with 50 μg/ml soluble PK followed by heating for 20 min at 100°C to deactivate the enzyme, and then prepared stimulant were treated in BMDMs. For pretreatment with polymyxin B (PMB; Sigma), LPS and Rv2145c were incubated in medium containing 50 μg/ml of PMB for 1 h at room temperature. After 24 h, cytokine levels in the supernatant were analyzed by sandwich ELISA.

### Anti-Rv2145c Antibody

BALB/c mice were immunized intraperitoneally three times with incomplete Freund’s adjuvant-emulsified Rv2145c (25 μg) at 2-week intervals. Serum collection was performed 1 week after the final immunization.

### Confocal Laser Scanning Microscopy

After the appropriate treatment or infection, the cells fixed on coverslips were permeabilized in 0.1% Triton X-100 for 10 min, incubated with primary antibodies for 24 h at 4°C, and then incubated with secondary antibodies for 1 h at room temperature. DAPI was used to stain nuclei. After mounting, immunofluorescence images were acquired using a confocal laser-scanning microscope (TCS SP8, Leica, Wetzlar, Germany) with consistent excitation, emission, pinhole, and exposure time parameters.

### Immunoprecipitation

Immunoprecipitation was performed as described previously (24). In brief, the cell lysate and 20 μg His-tagged Rv2145c (20 μg) were mixed and incubated at 4°C overnight, followed by mixing and precipitating with the relevant antibodies and Ni-NTA agarose (Qiagen, Hilden, Germany) beads. The beads were washed and the bound proteins were resolved by SDS-PAGE for immunoblotting with anti-TLR2 and anti-TLR4 antibodies.

### Immunoblotting Analysis

After the appropriate treatment or infection, the cell lysates were loaded onto SDS-polyacrylamide gels, and the separated protein were transferred to a nitrocellulose membrane. The membranes were blocked in 5% skim milk for 1 h at room temperature and then incubated with antibodies for 24 h at 4°C and subsequently with HRP-conjugated secondary antibodies for 1 h at room temperature. Target proteins were visualized using the ECL advance kit (GE Healthcare, Little Chalfont, UK).

### Indirect ELISA Analysis

Mtb was cultured in Sauton’s synthetic medium and harvested at 20 days and 56 days. The 5 μg of the culture filtrate was coated onto plates at 4°C for 24 h and then reacted with an anti-Rv2145c mouse antibody for 1 h, followed by an enzyme-conjugated secondary antibody. TMB substrate reagent (BD) was then added for color development. The plates were read on a Vmax kinetic microplate reader (Molecular Devices Co., Sunnyvale, CA, USA) at 450 nm.

### Subcellular Fractionation of Mtb

Mtb were grown in 7H9 medium supplemented with 0.5% glycerol and 10% OADC to an OD_600_ of 0.8. Cells were harvested by centrifugation, washed with DPBS, and lysed with lysis buffer containing 1 mM phenylmethylsulfonyl (PMSF), 5 mM DTT, 0.1 mg/ml lysozyme and protein cocktail inhibitor by sonication for 20 min. Lysates were centrifuged at 3,000 *g* for 30 min to remove unbroken cells. Whole-cell lysates were ultracentrifuged at 27,000 *g* for 30 min to separate and obtain the pellet fraction (cell wall) and supernatant (cytosol). The cell wall was washed with lysis buffer, recentrifuged, and resuspended in lysis buffer. All centrifugation steps were performed at 4°C.

### Transfections

BMDMs were transfected with above siRNA (siCON, 50 nM) and STAT3 siRNA (siSTAT3, 50 nM) using TurboFect according to the manufacturer’s instructions. After transfection, the cells were exposed to Mtb and Rv2145c, and then bacterial growth and cytokine production were analyzed.

### *In Vivo* Neutralization of IL-10 Receptor

The mice were injected intraperitoneally with 1 mg of either an anti-IL-10R antibody or IgG1 isotype control antibody and after 24 h, were infected intravenously with *M. smegmatis* (using 1 × 10^6^ CFU/mouse). After 24 or 72 h, the mice were sacrificed.

### Histopathology

Histopathology was performed as described previously (25). In brief, the prepared tissue sections were stained with hematoxylin and eosin (H&E) and acid-fast bacilli (AFB). All tissue analyses were performed through blind assessment. ImageJ software (National Institutes of Health, Bethesda, MD) was used to analyze histological image to quantify the granuloma area and bacterial count.

### Statistical Analysis

All performed experiments were repeated at least three times. Tukey’s multiple comparison test distribution or two-way ANOVA using statistical software (GraphPad Prism Software, version 4.03; GraphPad Software, San Diego, CA) were used to determine statistical significance between samples. Data in the graphs are presented as the mean values ± SD, and *p < 0.05, **p < 0.01 or ***p < 0.001 were considered statistically significant.

## Results

### Rv2145c Induces Macrophage Activation Through the TLR4 Pathway

**Figure 1:**
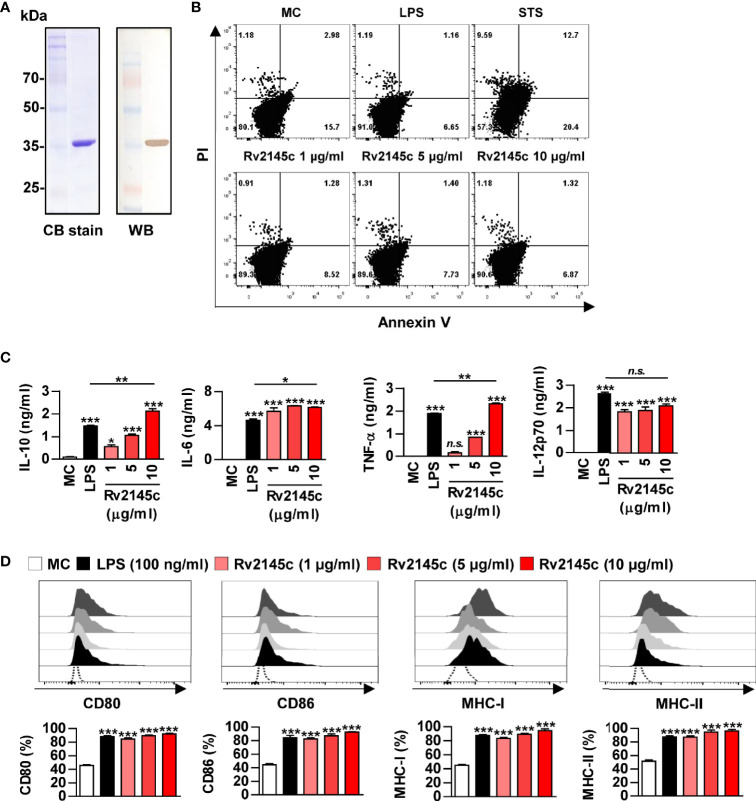
Recombinant Rv2145c induces macrophage activation. **(A)** Rv2145c purified from *E coli* extracts was analyzed by SDS-PAGE with Coomassie blue staining and immunoblot staining using an anti-His antibody. **(B)** The cytotoxic effects of Rv2145c were analyzed by flow cytometry. BMDMs were stimulated with Rv2145c (1, 5 or 10 μg/ml), LPS (100 ng/ml) or staurosporine (STS, 100 nM) for 24 h and then stained with Annexin V and PI. The percentage of positive cells in each quadrant is indicated. **(C, D)** BMDMs were stimulated with Rv2145c (1, 5 or 10 μg/ml) or LPS (100 ng/ml) for 24 h. IL-10, IL-6, TNF-α, and IL-12p70 levels in the culture supernatants were measured by ELISA **(C)**. The expression of surface markers was analyzed by two-color flow cytometry **(D)**. The cells were gated to exclude F4/80^+^ cells. BMDMs were stained with anti-CD80, anti-CD86, anti-MHC class I or anti-MHC class II antibodies. The bar graphs show the percentage (mean ± SD of three experiments) for each surface molecule on F4/80^+^ cells. All data are representative of three experiments. *p < 0.05, **p < 0.01, and ***p < 0.001 for treatment compared with medium controls (MC) or for the difference between treatment data. *n.s.*, no significant difference.

A recombinant Rv2145c protein purified from *Escherichia coli* (*E. coli*) appeared as a major band at 35 kDa on SDS-PAGE and reacted with an anti-His antibody (**Figure 1A**). Purified Rv2145c was not cytotoxic in bone marrow-derived macrophages (BMDMs) up to a concentration of 10 μg/ml (**Figure 1B**). We previously reported that MAP1889c, which is homologous with Rv2145c, stimulates DCs and macrophages to increase surface molecule presentation and secrete cytokines (23). Therefore, we investigated whether Rv2145c could also induce macrophage activation. As shown in **Figure 1C**, Rv2145c induced significant production of IL-10, IL-6, TNF-α, and IL-12p70 in a dose-dependent manner when compared to that in untreated BMDMs. At a concentration of 10 μg/ml, Rv2145c-stimulated BMDMs produced significantly higher levels of IL-10, IL-6, and TNF-α but not IL-12p70 than LPS (100 ng/ml)-stimulated BMDMs. The expression of co-stimulatory molecules and MHC class molecules in Rv2145c-treated BMDMs was significantly increased in a dose-dependent manner, which was comparable with that after LPS treatment (**Figure 1D**), when compared to that of the untreated controls. We next confirmed that Rv2145c-induced macrophage activation was not due to LPS contamination using heat denaturation or treatment with PK or PMB. The activity of Rv2145c was significantly decreased by boiling and PK treatment but not pretreatment with PMB (**Figure S1**). Taken together, these results suggest that Rv2145c induces macrophage activation with elevated IL-10, IL-6, and TNF-α, and moderate IL-12p70 production.

**Figure 2:**
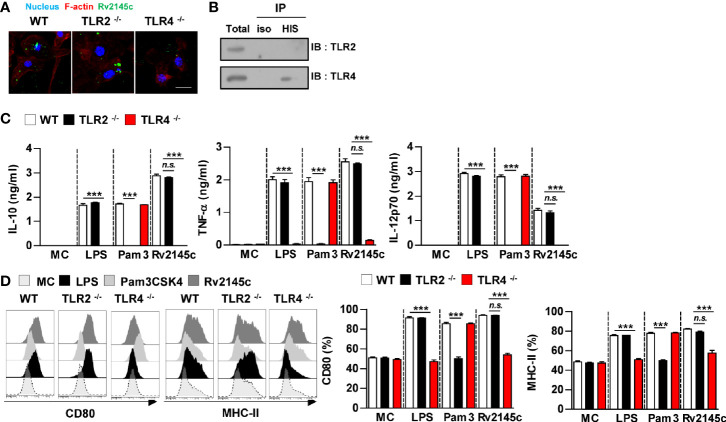
Rv2145c induces macrophage activation *via* TLR4 pathways. BMDMs derived from wild-type (WT), TLR2^–/–^, and TLR4^–/–^ mice were treated with Rv2145c (10 μg/ml), LPS (100 ng/ml), or Pam3CSK4 (Pam3) (100 ng/ml) for 24 h. **(A)** BMDMs-treated with Rv2145c for 1 h were fixed and then stained with DAPI (blue) and an Alexa Fluor 488-conjugated anti-Rv2145c antibody. Representative images from three independent experiments are shown. Scale bar, 10 μm. **(B)** The lysates from BMDMs treated with Rv2145c for 6 h were used for immunoprecipitation with anti-mouse IgG or anti-His antibodies. Thereafter, proteins were detected using immunoblotting with anti-TLR2 or anti-TLR4 antibodies. The total is shown as the mean total in cell lysates (input). **(C)** The production of IL-10, TNF-α, and IL-12p70 in the culture supernatants was determined by ELISA. All data are expressed as the mean ± SD (*n* = 3). **(D)** Expression of CD80, and MHC class II molecules on BMDMs stimulated with each antigen was determined by staining and flow cytometry. The bar graphs show the mean percentage ± SD of each surface molecule on F4/80^+^ cells across three independent experiments. ***p < 0.001 for treatment values in BMDMs from TLR2^-/-^ or TLR4^-/-^ mice compared with those in Rv2145c-, LPS- or Pam3CSK4-treated BMDMs from WT mice. *n.s.*, no significant difference. MC, medium controls.

We next determined whether Rv2145c stimulated BMDMs through TLR recognition. Confocal microscopy showed that Rv2145c interacted with the surface of BMDMs from WT and TLR2^-/-^ BMDMs but did not bind to TLR4^-/-^ BMDMs (**Figure 2A**). Immunoprecipitation analysis with an anti-His antibody also showed that Rv2145c directly interacted with TLR4 molecules (**Figure 2B**). Furthermore, Rv2145c-mediated cytokine production and surface molecule expression were significantly depressed in TLR4^-/-^ BMDMs compared to those in WT and TLR2^-/-^ BMDMs, while LPS and Pam3 activities were decreased in TLR4^-/-^ BMDMs and TLR2^-/-^ BMDMs, respectively (**Figures 2C, D**). These results suggest that Rv2145c activated macrophages *via* TLR4 pathway.

### Rv2145c Induces Macrophage Activation Through the STAT3, MAPK, and NF-κB pathways

**Figure 3:**
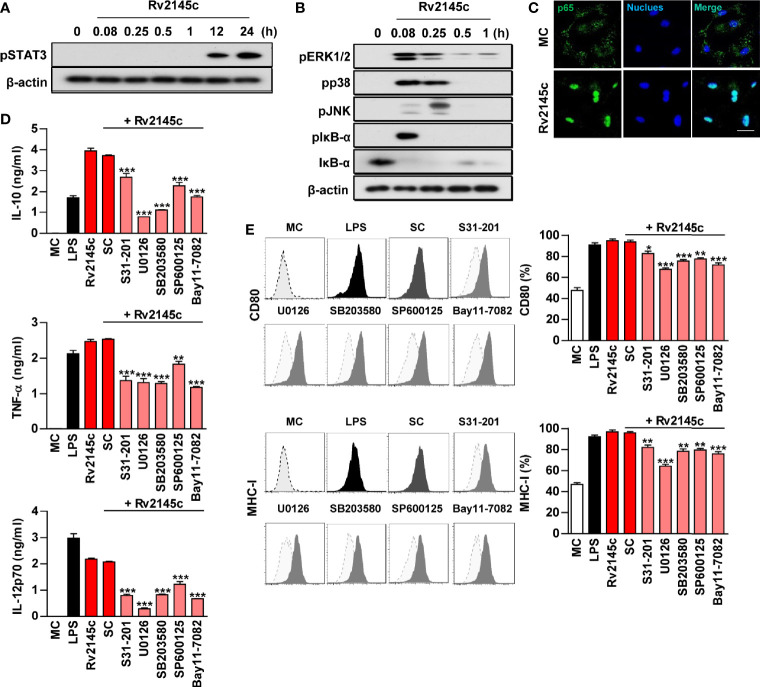
Rv2145c induces macrophage activation *via* STAT3, MAPK, and NF-κB. BMDMs stimulated with Rv2145c for the indicated times were lysed, and the proteins in the total cell lysate were separated by SDS-PAGE followed by immunoblot analysis using antibodies against **(A)** phospho-STAT3 and **(B)** phospho-ERK1/2, phospho-p38, phospho-JNK, phospho-IκB-α, IκB-α, and β-actin. This image is representative of three experiments showing similar results. **(C)** BMDMs were plated in covered glass chamber slides and treated with Rv2145c for 1 h, and the immunoreactivity of the p65 subunit of NF-κB in cells was determined by immunofluorescence. Scale bar, 10 μm. **(D, E)** BMDMs were pretreated with pharmacological inhibitors of STAT3 (S31-201, 5 μM), ERK (U0126, 10 μM), p38 (SB203580, 20 μM), JNK (SP600125, 10 μM), NF-κB (BAY11-7082, 5 μM), or DMSO (SC; solvent control) for 1 h prior to treatment with Rv2145c (10 μg/ml). After 24 h, the amounts of IL-10, TNF-α, and IL-12p70 in the culture medium were measured by ELISA **(D)**. The mean ± SD is shown for three independent experiments. The expression levels of CD80 and MHC-I were analyzed by flow cytometry **(E)**. Bar graphs show percentages (mean ± SD of three separate experiments) for each surface molecule on F4/80^+^ cells. *p < 0.05, **p < 0.01, or ***p < 0.001 for each inhibitor treatment compared with Rv2145c-treated controls. MC, medium controls; SC, solvent controls.

We analyzed whether Rv2145c induced macrophage activation *via* STAT3, MAPKs and NF-κB. As shown in **Figures 3A, B**, Rv2145c triggered strong phosphorylation of STAT3, ERK1/2, p38 and JNK and phosphorylation and degradation of IκB-α in BMDMs. STAT3 was activated later than MAPKs. In addition, a confocal assay showed that Rv2145c induced robust nuclear translocation of p65 from the cytosol (**Figure 3C**). To confirm the functional roles of STAT3, MAPK and NF-κB signaling in Rv2145c-mediated macrophage activation, pharmacological inhibitors were pretreated before Rv2145c stimulation. Predictably, these inhibitors significantly suppressed Rv2145c-mediated IL-10, TNF-α, and IL-12p70 production (**Figure 3D**) and expression of surface molecules such as CD80 and MHC-I (**Figure 3E**). Our data indicate that the STAT3, MAPKs and NF-κB signaling pathways are involved in Rv2145c-mediated macrophage activation.

### Rv2145c Is Mainly Located on the Cell Wall and Enhances Intracellular Mtb Growth

**Figure 4:**
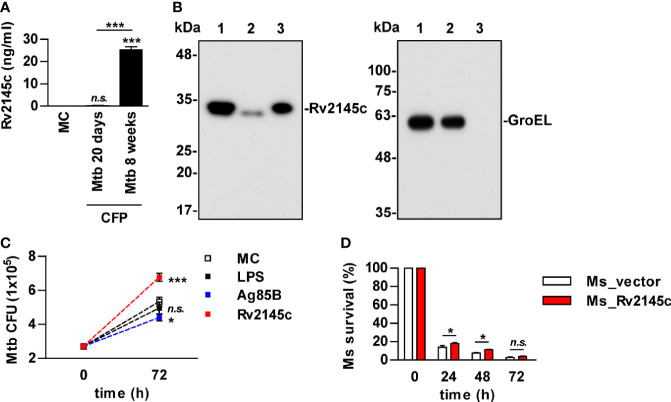
Rv2145c is mainly present in the mycobacterial cell wall and promotes bacterial growth in macrophages. **(A)** Mtb was cultured in Sauton’s medium for 20 days and 8 weeks. The level of Rv2145c in culture filtrate proteins (CFP) was measured by indirect-ELISA. CFP was added to a plate, and then a mouse anti-Rv2145c antibody was added. HRP-conjugated anti-mouse IgG was then added, and TMB substrate was added and converted by HRP before detection. **(B)** Rv2145c in fractions from Mtb was detected by Western blot analysis using an anti-His antibody. The expression of cytosolic GroEL was detected by anti-GroEL antibody as a positive control. Lane 1: whole-cell lysate; lane 2: cytosolic fraction; lane 3: cell wall fraction. **(C)** BMDMs were infected with Mtb at a multiplicity of infection (MOI) of 1 for 4 h, further treated with gentamicin to kill extracellular bacteria for 2 h, and incubated with or without 10 μg/ml Rv2145c, 100 ng/ml LPS or 5 μg/ml Ag85B for 72 h. Intracellular bacterial growth was determined by plating the cell lysates on 7H10 agar. **(D)** BMDMs were infected with *M. smegmatis* expressing Rv2145c (Ms_Rv2145c) or a vector control strain (Ms_vector) at an MOI of 10 for 4 h and further treated with gentamicin to kill extracellular bacteria for 2 h. The medium was then changed. CFU assays were conducted at the indicated times. The mean ± SD is shown for three independent experiments. *p < 0.05 and ***p < 0.001 for the treatment compared with MC or for the difference between the treatment or vector controls (Ms_vector). *n.s.*, no significant difference. MC, medium controls.

It has been reported that Rv2145c is related to cell wall and cell processes in functional categories and is associated with the cell membrane fraction (26, 27). We also found that Rv2145c was detected in the supernatants of *Mycobacterium tuberculosis* (Mtb) cultured for 8 weeks but not 3 weeks (**Figure 4A**). Immunoblot assays showed that Rv2145c was located mostly in the cell wall fraction of Mtb but was also weakly detected in the cytosol fraction (**Figure 4B**). GroEL was also detected as an indicator of the cytosolic fraction. As described above, Rv2145c induced relatively higher IL-10 production in macrophages, and some mycobacterial proteins involved in cell wall biogenesis are responsible for Mtb virulence and contribute to its ability to grow in macrophages (28, 29). Therefore, we determined whether Rv2145c could affect Mtb growth in macrophages. Mtb-infected BMDMs were stimulated with LPS, Rv2145c, and Ag85B, which were used as unrelated control Mtb antigens, and then intracellular survival was determined. As shown in **Figure 4C**, intracellular Mtb growth was significantly higher in Rv2145c-stimulated BMDMs than in untreated or LPS- or Ag85B-treated BMDMs. In addition, Rv2145c-mediated enhancement of intracellular Mtb growth was observed in BMDMs from WT and TLR2^-/-^ mice but not in BMDMs from TLR4^-/-^ mice (**Figure S2**). Next, we tested whether Rv2145c basically expressed in the Mtb cell wall could affect bacterial survival by using an anti-Rv2145c antibody. Intracellular Mtb growth was significantly reduced in BMDMs pretreated with the anti-Rv2145c antibody before Mtb infection compared with that in untreated or isotype control-treated cells (**Figure S3**). To further confirm the role of Rv2145c in bacterial survival, Rv2145c was overexpressed in nonpathogenic *Mycobacterium smegmatis* (*M. smegmatis*) by using the pVV16 shuttle vector. We confirmed that the recombinant *M. smegmatis* strain expressed Rv2145c, and there was no significant difference *in vitro* growth rates between the Rv2145c-expressing recombinant strain (Ms_Rv2145c) and the vector control strain (Ms_vector) (**Figure S4**). The vector control *M. smegmatis* was more rapidly cleared in macrophages than the Rv2145c-expressing strain (**Figure 4D**). Taken together, these data suggest that Rv2145c may play an important role in Mtb survival in macrophages.

### Rv2145c Enhances STAT3 Activation in Mtb-Infected Macrophages

**Figure 5:**
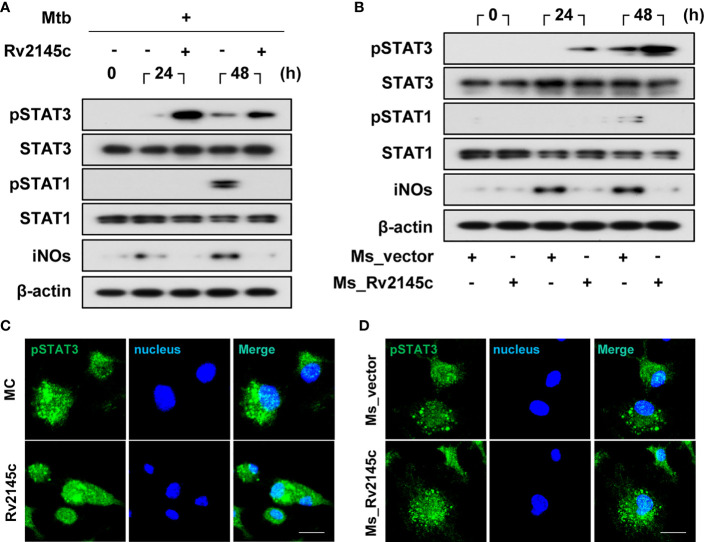
Rv2145c induces activation of STAT3 in Mtb-infected macrophages. **(A, B)** BMDMs were infected with **(A)** Mtb at an MOI of 1 for 4 h and incubated with or without 10 μg/ml Rv2145c or **(B)** Ms_vector or Ms_Rv2145c at an MOI of 10 for 4 h. The cells were lysed at the indicated times, and the proteins in the total cell lysate were separated by SDS–PAGE, followed by immunoblot analysis using antibodies against phospho-STAT3, STAT3, phospho-STAT1, STAT1, iNOs and β-actin. This image is representative of three experiments showing similar results. **(C, D)** At 48 h after Mtb infection **(C)** or at 24 h after *M. smegmatis* infection **(D)**, BMDMs were fixed with 4% paraformaldehyde and immunolabeled with an anti-phospho-STAT3 antibody, followed by Alexa 488-conjugated goat anti-rabbit IgG. The cells were stained with DAPI to visualize the nuclei (blue). The localization of the target molecules was analyzed by laser-scanning confocal microscopy. Scale bar, 10 μm. MC, medium controls.

STAT3 pathways are involved in the survival of Mtb in macrophages (14), and Rv2145c induced the phosphorylation of STAT3 from 12 h after stimulation in BMDMs (**Figure 3A**). Therefore, we investigated the effects of Rv2145c on STAT3 phosphorylation in macrophages during Mtb infection. As shown in **Figure 5A**, phosphorylation of STAT3 was weakly induced in BMDMs infected with Mtb at 24 and 48 h but was strongly enhanced by Rv2145c treatment. STAT1 is required for intracellular signaling and cellular responses to IFN-γ, and IFN-γ-mediated NO production is an intracellular Mtb-killing mechanism in macrophages (30, 31). Rv2145c did not enhance STAT1 activation and iNOs expression, but suppressed STAT1 activation and iNOs expression mediated by Mtb at 48 h (**Figure 5A**). *M. smegmatis* expressing Rv2145c also strongly induced activation of STAT3 but not STAT1 activation and iNOs expression compared to those with the vector control strain (**Figure 5B**). In addition, confocal microscopy assays showed that nuclear translocation of phosphorylated STAT3 in Mtb-infected BMDMs was increased by Rv2145c treatment (**Figure 5C**), and this translocation was also higher in cells infected with Rv2145c-expressing *M. smegmatis* than in cells infected with the vector control strain (**Figure 5D**).

### STAT3 Is Required for Rv2145c-Mediated Intracellular Survival and IL-10 Production

**Figure 6:**
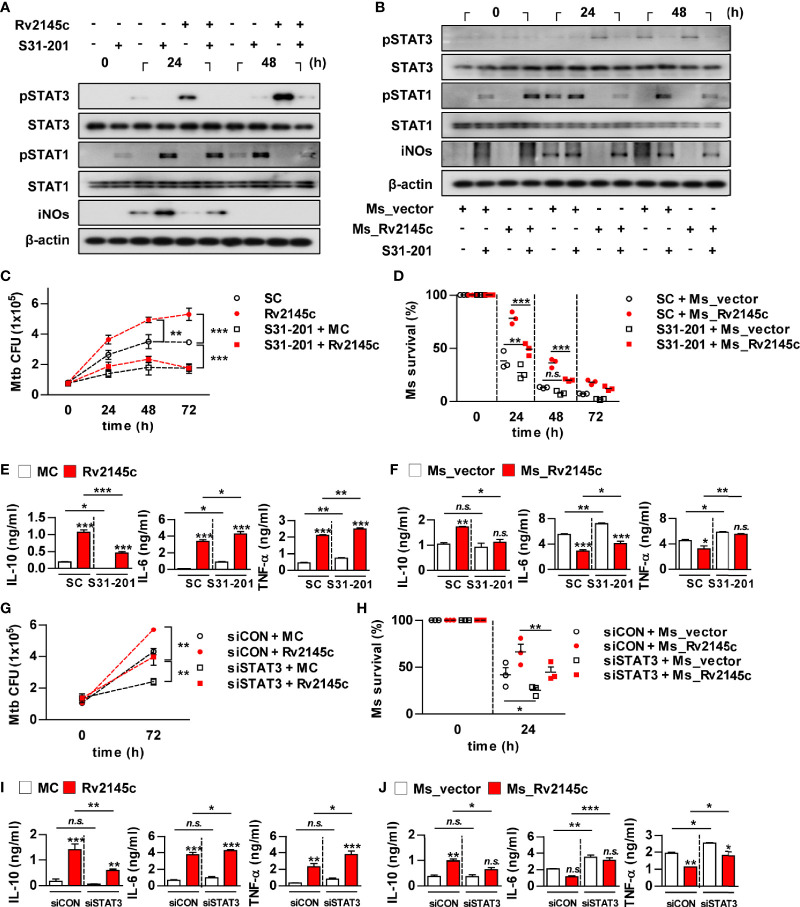
STAT3 signaling is important for Rv2145c-mediated intracellular survival and IL-10 production. **(A-F)** BMDMs pretreated with pharmacological inhibitors of STAT3 (S31-201, 5 μM) for 1 h were infected with **(A, C, E)** Mtb at an MOI of 1 for 4 h and treated with 10 μg/ml Rv2145c or **(B, D, F)** Ms_vector or Ms_Rv2145c at an MOI of 10 for 4 h. Then, the cells were cultured in the presence and absence of S31-201 for the indicated times. **(A, B)** Total cell lysates were separated by SDS–PAGE, followed by immunoblot analysis using antibodies against phospho-STAT3, STAT3, phospho-STAT1, STAT1, iNOs and β-actin. This image is representative of three experiments showing similar results. **(C, D)** Bacterial CFUs were determined at the indicated times. **(E, F)** IL-10, IL-6, and TNF-α levels in the culture supernatants at 72 h **(E)** or 24 h **(F)** were measured by ELISA. **(G–J)** BMDMs were transfected with STAT3 siRNA (siSTAT3) or non-specific siRNA as a control (siCON) and were infected with **(G, I)** Mtb at an MOI of 1 for 4 h, and incubated with 10 μg/ml Rv2145c or **(H, J)** Ms_vector or Ms_Rv2145c at an MOI of 10 for 4 h, incubated for the indicated times. **(G, H)** Intracellular bacterial growth was determined by plating the cell lysates on 7H10 agar. **(I, J)** IL-10, IL-6, and TNF-α levels in the culture supernatants at 72 h **(I)** or 24 h **(J)** were measured by ELISA. *p < 0.05, **p < 0.01, and ***p < 0.001 for treatment compared with medium controls (MC) or for the difference between treatment data. *n.s.*, no significant difference. SC, solvent controls.

We next investigated whether Rv2145c-mediated STAT3 activation could affect Mtb growth in macrophages. As expected, a STAT3 inhibitor (S31-201) completely suppressed Rv2145c-mediated STAT3 phosphorylation and enhanced the phosphorylation of STAT1 and iNOs expression in Mtb-infected macrophages (**Figure 6A**). Similarly, S31-201 inhibited STAT3 activation and enhanced STAT1 activation and iNOs expression in macrophages infected with Rv2145c-expressing *M. smegmatis*, and the same reactive pattern was observed in macrophages infected with the vector control strain (**Figure 6B**). Pretreatment with S31-201 significantly suppressed intracellular Mtb growth compared to that with the DMSO control (SC; solvent control), abrogated Mtb growth enhancement induced by Rv2145c (**Figure 6C**), and more prominently inhibited the growth of Rv2145c-expressing *M. smegmatis* than the vector control strain in macrophages (**Figure 6D**). Rv2145c induced significant IL-10, IL-6, and TNF-α production in Mtb-infected macrophages when compared to that in Mtb only-infected cells (**Figure 6E**). Interestingly, pretreatment S31-201 significantly inhibited Rv2145c-mediated IL-10 production but enhanced Rv2145c-mediated IL-6 and TNF-α production compared to those in untreated cells (**Figure 6E**). BMDMs infected with Rv2145c-expressing *M. smegmatis* produced significantly higher IL-10 and lower IL-6 and TNF-α than vector control strain-infected cells, and pretreatment with S31-201 significantly inhibited IL-10 production induced by Rv2145c-expressing *M. smegmatis*, but enhanced IL-6 and TNF-α production when compared to that of untreated BMDMs (**Figure 6F**). There was no difference in IL-10 production induced by the vector control between S31-201-treated and untreated cells. To further confirm the role of STAT3, BMDMs transfected with siSTAT3 were infected with Mtb and then stimulated with Rv2145c. We first confirmed that Mtb- or Rv2145c-induced STAT3 activation in Mtb-infected macrophages was abrogated by transfection with siRNA specific to STAT3 (siSTAT3) (**Figure S5**). Similar to STAT3 inhibition, STAT3 gene silencing led to a significant reduction in Mtb growth compared to that in siCON-transfected cells, abrogation of Rv2145c-mediated enhancement of Mtb growth (**Figure 6G**), and more rapid clearance of Rv2145c-expressing *M. smegmatis* compared to that in siCON-transfected cells (**Figure 6H**). In addition, transfection of siSTAT3 resulted in a significant reduction in Rv2145c-mediated IL-10 production and enhancement of Rv2145c-mediated IL-6 and TNF-α production in Mtb-infected BMDMs (**Figure 6I**). The same patterns in cytokine production induced by Rv2145c-expressing *M. smegmatis* were observed by transfection with siSTAT3 (**Figure 6J**). Taken together, these results suggest that STAT3 activation is required for Rv2145c-mediated Mtb growth enhancement.

### Rv2145c Enhances Intracellular Mtb Growth *via* IL-10 Receptor Signaling

**Figure 7:**
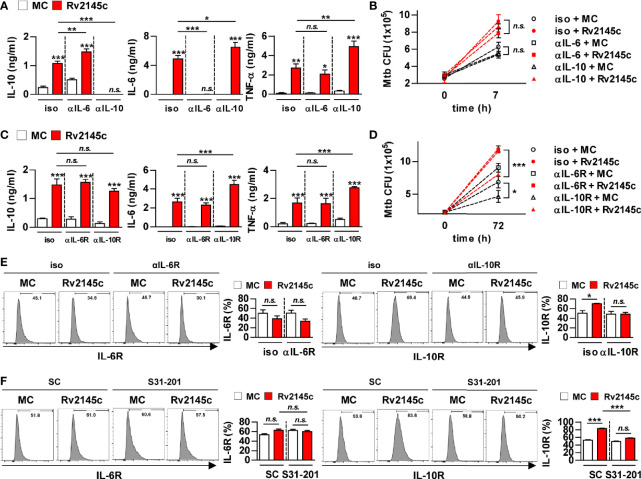
Rv2145c enhances intracellular Mtb growth *via* IL-10 receptor signaling. **(A-E)** BMDMs were pretreated with anti-IL-6 (αIL-6, 5 μg/ml) antibody, anti-IL-10 (αIL-10, 5 μg/ml) antibody, mouse control IgG (iso, 5 μg/ml) for cytokine neutralization or anti-IL-6R (αIL-6R, 1 μM), anti-IL-10R (αIL-10R, 1 μM) or mouse control IgG (iso, 1 μM) for receptor blocking, and then BMDMs were infected with Mtb at an MOI of 1 for 4 and treated with 10 μg/ml Rv2145c. Then, the cells were cultured in the presence of each neutralizing antibody or receptor blocking antibody for 72 h. **(A, C)** IL-10, IL-6, and TNF-α levels in the culture supernatants were measured by ELISA. **(B, D)** Intracellular Mtb growth was determined by plating the cell lysates on 7H10 agar at 0 h and 72 h. **(E)** The expression of receptor markers was analyzed by two-color flow cytometry. The cells were gated to exclude F4/80^+^ cells. BMDMs were stained with anti-IL-6R or anti-IL-10R antibodies. **(F)** BMDMs pretreated with inhibitors of STAT3 (S31-201, 5 μM) for 1 h and then infected with Mtb at an MOI of 1 for 4 h and treated with 10 μg/ml Rv2145c, and then the cells were incubated in the presence and absence of S31-201 for 72 h. The expression of the receptor markers was analyzed by two-color flow cytometry. The cells were gated to exclude F4/80^+^ cells. BMDMs were stained with anti-IL-6R or anti-IL-10R antibodies. The bar graphs show the percentage (mean ± SD of three experiments) for each surface molecule on F4/80^+^ cells. *p < 0.05, **p < 0.01, and ***p < 0.001 for treatment compared with medium controls (MC) or for the difference between treatment data. *n.s.*, no significant difference.

Because STAT3 inhibition resulted in a significant reduction in Rv2145c-mediated IL-10 production and an increase in Rv2145c-mediated IL-6 and TNF-α production, we next investigated the role of IL-10 in Rv2145c-induced Mtb growth enhancement. First, we determined the neutralizing effect of IL-10 on Rv2145c activity. Mtb-infected BMDMs were pretreated with anti-IL-6 or anti-IL-10 antibodies prior to adding Rv2145c. IL-10 and IL-6 induced by Rv2145c were undetected after pretreatment with anti-IL-10 and anti-IL-6 antibodies, respectively, and Rv2145c-mediated IL-6 and TNF-α production was significantly increased by pretreatment with the anti-IL-10 antibody in Mtb-infected BMDMs, whereas Rv2145c-mediated IL-10 production was significantly increased by pretreatment with the anti-IL-6 antibody (**Figure 7A**). However, anti-IL-10 or anti-IL-6 antibodies did not affect intracellular Mtb growth or enhance Rv2145c-mediated Mtb growth in BMDMs (**Figure 7B**). Next, we hypothesized that Rv2145c might regulate the IL-10 receptor (IL-10R) or IL-6 receptor (IL-6R) signaling pathways. The anti-IL-10R antibody did not affect Rv2145c-mediated IL-10 production in Mtb-infected BMDMs but induced a significant increase in Rv2145c-mediated IL-6 and TNF-α production compared to that in isotype-treated cells (**Figure 7C**). The anti-IL-6R antibody did not induce any change in Rv2145c-mediated cytokine production. Interestingly, the anti-IL-10R antibody, but not anti-IL-6R antibody, significantly inhibited intracellular Mtb growth and abrogated Rv2145c-mediated Mtb growth enhancement (**Figure 7D**). These results suggest that the increase in intracellular bacterial survival induced by Rv2145c is closely related to IL-10R signaling in macrophages. Therefore, we next investigated the effect of Rv2145c on the expression of cytokine receptors in Mtb-infected macrophages. Rv2145c induced a significant increase in the expression of IL-10R, which was abolished by pretreatment with the anti-IL-10R antibody (**Figure 7E**). However, Rv2145c did not affect IL-6R expression in Mtb-infected macrophages. Similarly, the STAT3 inhibitor abolished the enhancement of Rv2145c-mediated IL-10R expression (**Figure 7F**). There was no difference in the expression of IL-6R regardless of the presence or absence of S31-201. These results indicated that Rv2145c-mediated IL-10R expression *via* the STAT3 pathway leads to the enhancement of Mtb growth in macrophages.

### Approximately Two-Thirds of the Rv2145c C-Terminus Is Functionally Active in Modulating Host Cell Signaling

Rv2145c (Wag31) is a homolog of DivIVA (a cell division and cell shape protein), which regulates cell morphology in gram-positive bacteria (32) and contains two coiled-coil domains on the N-terminal side (32-59 aa) and C-terminal side (166-193 aa), a DivIVA functional site (3-63 aa), and an N-terminal-membrane binding domain. Therefore, we produced two truncated Rv2145c proteins: the N-terminal part (1-90 aa, domain 1; D1) and the C-terminal part (91-260 aa, domain 2; D2) (**Figure S6A**). Domain 2 of Rv2145c induced significantly higher IL-10, IL-6 and TNF-α production than domain 1 of Rv2145c and produced amounts comparable to those of full-length Rv2145c protein, at a concentration of 10 μg/ml (**Figure S6B**). We also confirmed that there was no endotoxin contamination in the purified recombinants (**Figure S6C**). Furthermore, domain 2 but not domain 1 significantly enhanced intracellular Mtb growth in BMDMs compared to that in the medium control (**Figure S6D**). These results suggest that the DivIVA functional active site that regulates cell morphology is not involved in the modulation of intracellular survival mediated by Rv2145c.

### Rv2145c Expression Induces an Increase in *M. smegmatis* Virulence *In Vivo*

**Figure 8:**
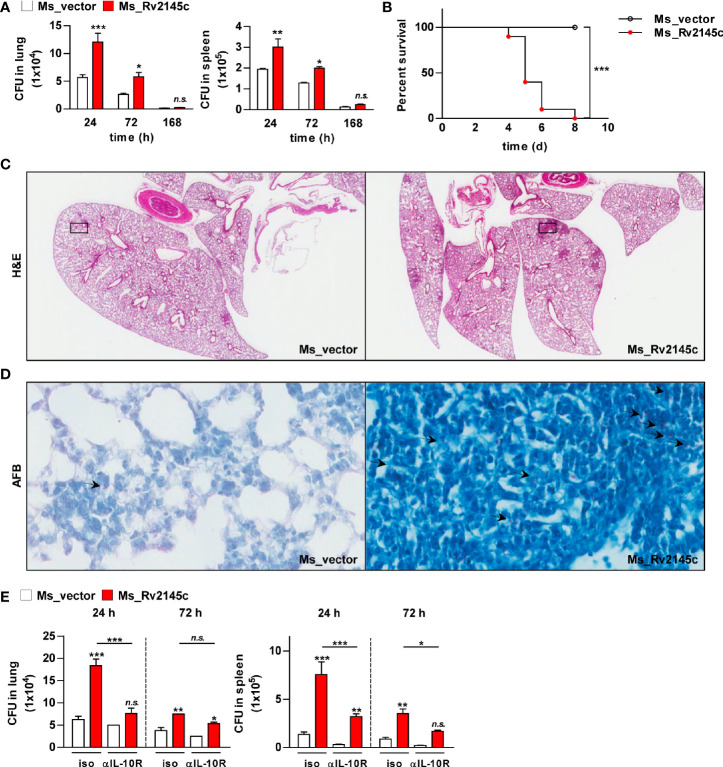
Expression of Rv2145c increases virulence of *M. smegmatis in vivo*. **(A)** C57BL/6 mice (n = 3 per group at each time point) were infected intravenously with Ms_vector or Ms_Rv2145c (1 × 10^6^ CFU/mouse). Mice were sacrificed at the indicated time points (24, 72 and 168 h), CFUs were determined in the lungs and spleens. **(B)** For the survival test, C57BL/6 mice (n = 5 per group) were infected intravenously at a high dose (1 × 10^7^ CFU/mouse). **(C, D)** The lung tissues of Ms_vector or Ms_Rv2145c strain (1 × 10^6^ CFU/mouse)-infected mice were collected at 72 h post infection and stained with **(C)** hematoxylin and eosin (H&E) and for **(D)** acid-fast bacilli (AFB). (H&E magnification ×10, AFB magnification ×400). The granuloma area and the bacterial count from a lung section were plotted. **(E)** C57BL/6 mice (n = 3 per group at each time point) were treated with 1 mg/ml anti-IL-10R antibody or isotype control antibody intraperitoneally, and the next day, they were infected intravenously at a dose of 1 × 10^6^ CFU/mouse. Mice were sacrificed at the indicated time points (24 and 72 h), and CFUs were determined in the lungs and spleens. The mean ± SD is shown for three independent experiments. *p < 0.05, **p < 0.01 and ***p < 0.001 compared vector controls or difference between treatment data. Treatments with no significant effect are indicated as *n.s.*

To estimate the role of Rv2145c during bacterial infection, the mice were infected with Rv2145c-expressing *M. smegmatis* and vector control strains. As shown in **Figure 8A**, clearance of Rv2145c-expressing *M. smegmatis* in the lung and spleen was significantly delayed compared with that of the vector control strain. All mice infected with the vector control strain survived, but all mice infected with Rv2145c-expressing *M. smegmatis* died by 8 days post-infection (**Figure 8B**). Histopathologic findings showed that pathologic lesions were prominently more severe in the lungs of mice infected with Rv2145c-expressing *M. smegmatis* than in those of mice infected with the vector control strain (**Figure 8C**), and AFB counts were higher in tissue sections of lungs infected with Rv2145c-expressing *M. smegmatis* than in those of the vector control strain-infected mice (**Figure 8D**).

Finally, we tested whether IL-10R signaling was involved in Rv2145c-mediated bacterial control in the *in vivo* system. The bacterial loads in the lungs and spleens of the mice infected with Rv2145c-expressing *M. smegmatis* were significantly reduced by administration of an anti-IL-10R antibody compared with those in the mice injected with an isotype antibody (**Figure 8E**). The growth of the *M. smegmatis* vector control in mice injected with the anti-IL-10R antibody was lower than that in mice injected with the isotype antibody, but there was no significant difference between the groups. Overall, these results indicate that overexpression of Rv2145c makes nonpathogenic mycobacteria more virulent in mice and that IL-10R signaling is intimately related.

## Discussion

Understanding the functional proteins that play a pathogenic role during Mtb infection is essential for the comprehension of host-pathogen interactions and identification of vaccine candidates. In fact, some proteins of the PE/PPE family that are able to modulate the immune response have been used in multi-subunit TB vaccines (33). In this study, we reported the enhancing mechanism of Mtb growth by Rv2145c protein that strongly stimulated IL-10 production, suggesting that this protein may be a new virulence factor and supporting a role of IL-10R and STAT3 signaling during Mtb infection.

Rv2145c, which is known as Wag31 or antigen 84, is located in the cell wall fraction of bacteria (34) and plays a critical role in cell division (35). Transposon mutagenesis studies have shown that *Rv2145c* is an essential gene for the *in vitro* growth of Mtb (36, 37). Rv2145c is present in most mycobacteria, and its roles in cell wall synthesis and morphology have been demonstrated (38–40), but its immunobiological function is mostly unknown. We previously reported that *Mycobacterium avium* subsp. *paratuberculosis* MAP1889c, which has homology with Rv2145c, induces DC maturation and Th2 responses and promotes *Mycobacterium avium* growth and IL-10 production in macrophages (23). In the present study, Rv2145c induced macrophage activation with elevated IL-10 production (**Figure 1**). Rv2145c has relatively conserved sequences in both N-terminal and C-terminal regions among the mycobacteria (41). In this study, the N-terminal part of Rv2145c was not involved in Rv2145c-mediated IL-10 production and Mtb growth enhancement. Samten B et al. reported that Wag31 and its C-terminal domain inhibit anti-CD3 and anti-CD28 antibody-mediated IL-10 and IL-17 production in human T cells (41), but this study was performed in purified T cells without antigen-presenting cell involvement.

Compared with LPS, Rv2145c induced significant production of IL-10, IL-6, and TNF-α but not IL-12p70 in macrophages. It is known that the development of Th1 and Th2 responses is dependent on IL-12 or IL-10 production by macrophages or dendritic cells activated with mycobacteria and their components. Several Mtb proteins that induce Th2 immune responses have been reported; the PE25/PPE41 protein complex induces DC maturation and the production of IL-1β, IL-10 and TNF-α but not IL-12p70, resulting in Th2-biased responses (42), and Rv1917c (PPE34)-stimulated DCs secrete high levels of IL-6, TNF-α and IL-10 but not IL-12 and induce Th2 cytokine responses (21). PPE18 stimulates macrophages to secrete IL-10 and skews responses toward the Th2 type (22). Although IL-10 induced by Mtb and/or its components contributes to bacterial survival, very little is known about how these proteins inducing IL-10 production can directly modulate the Mtb growth in macrophages. Therefore, in this study, we determined whether Rv2145c could affect intracellular Mtb growth. Rv2145c treatment led to significant enhancement of Mtb growth in macrophages, and its expression in *M. smegmatis* resulted in delayed clearance by macrophages (**Figure 4**). However, neutralization of IL-10 or IL-6 with antibodies did not affect the enhancement of Rv2145c-mediated Mtb growth; instead, blocking IL-10R significantly inhibited intracellular Mtb growth and abrogated Rv2145c-mediated Mtb growth enhancement. These results suggest that receptor blocking is more effective and susceptible to interfere with Rv2145c-mediated function. In addition, Rv2145c induced the expression of IL-10R in Mtb-infected macrophages.

IL-10-STAT3 signaling is essential for the regulation of the immune response as a potent immunomodulatory pathway, but additionally inhibits the bactericidal capacity of macrophages. In this study, Rv2145c induced the activation of STAT3 in both uninfected macrophages and Mtb-infected macrophages. STAT3 inhibition by a chemical inhibitor and siRNA induced a significant reduction in Mtb growth and abrogation of Rv2145c-mediated enhancement of Mtb growth. In addition, blocking STAT3 signaling led to decreased IL-10 production, increased production of IL-6 and TNF, and decreased IL-10R expression. During Mtb infection, IL-10 activates STAT3 signaling in infected and surrounding cells (14). Therefore, our data suggest that STAT3 is initially activated by IL-10 produced by Rv2145c and that IL-10 production and IL-10R expression are amplified by activated STAT3, subsequently resulting in the promotion of Mtb replication. Jaslow SL et al. also reported that STAT3 signaling is required for intracellular *Salmonella* replication and IL-10 production (43). Macrophages from STAT3-deficient mice have been shown to increase the production of inflammatory cytokines such as TNF and IL-6 and antigen-presenting molecules (44, 45). In addition, in this study, Rv2145c suppressed Mtb-mediated STAT1 activation and iNOs expression (**Figure 5**). *M. smegmatis* expressing Rv2145c showed similar patterns with IL-10-STAT3 responses and functions induced by Rv2145c treatment in macrophages. Taken together, our data suggest that Rv2145c plays a role in creating a favorable environment for bacterial survival by modulating host signals.

Numerous papers have demonstrated that mycobacterial proteins induce the activation of macrophages or dendritic cells *via* the TLR2 or TLR4 pathway. Most PE/PPE proteins interact with the TLR2 molecule, but PE9-PE10 interacts with TLR4 (46), while other proteins, such as Rv2882 (47), Rv3463 (24), Rv2299c (48), and MAP1889c (23), interact with TLR4. In this study, Rv2145c induced macrophage activation by interacting with TLR4 (**Figure 2**). In addition, Rv2145c-mediated Mtb growth enhancement was not observed in the BMDMs from TLR4^-/-^ mice.

It has been reported that Rv2145c is present in the cell wall fraction of Mtb (49). In this study, Rv2145c was also mostly present in the cell wall fraction. Mtb contains complex components that are able to induce pro- and anti-inflammatory responses. In fact, in this study, phosphorylation of both STAT1 and STAT3 was observed in macrophages infected with Mtb only. In this study, because Rv2145c was additionally added to Mtb-infected macrophages in our assay system, it is possible that Rv2145c activities, such as Mtb growth enhancement, may be overinterpreted. Therefore, to confirm the exact role of Rv2145c, the Mtb mutant strain in which the *Rv2145c* gene is deleted is required, but this gene is an essential gene. We thus performed similar experiments by using *M. smegmatis* expressing Rv2145c. Its intracellular survival was increased, which was abrogated by STAT3 inhibition and IL-10R blockade. Furthermore, clearance of Rv2145c-expressing *M. smegmatis* in the lung and spleen of mice was delayed, but these effects were abrogated by administration of anti-IL-10R antibody before infection. When mice were infected with a higher dose, all mice infected with Rv2145c-expressing *M. smegmatis* died, whereas all mice infected with the vector control survived. These studies clearly demonstrate another virulence mechanism by which Mtb can use host-derived factors to establish chronic infection through subversion of the protective host response.

Although Rv2145c is mainly located in the mycobacterial cell wall fraction, it has also been identified in culture supernatants (50). In the present study, it was detected in late culture filtrate but not early culture filtrate of Mtb (**Figure 4**), indicating that Rv2145c is released into the medium by shearing from the cell wall. It has been demonstrated that the expression of IL-10 and STAT3 is increased in cells located at the infected site in the lungs of mice chronically infected with Mtb (15, 51). In these respects, we speculated that Rv2145c released into surrounding cells from chronically infected Mtb may play a pathogenic role by promoting the expression of IL-10/IL-10R and the activation of STAT3. In addition, Rv2145c is one of the 451 drug targets identified by the *in silico* target TB pipeline (52). Therefore, together, our findings suggest that Rv2145c deserves more in-depth study especially focused on designing potential drugs and vaccine candidates against TB.

## Data Availability Statement

The datasets presented in this study can be found in online repositories. The names of the repository/repositories and accession number(s) can be found in the article/**Supplementary Material**.

## Ethics Statement

The animal study was reviewed and approved by Institutional Animal Care and Use Committee of Chungnam National University, Daejeon, South Korea (permit number: CNU-01043).

## Author Contributions

H-SP and H-JK designed the study. H-SP, YB, I-TJ, K-IL, Y-JS, H-GC and TD performed experiments and analyzed data. H-JK supervised the work and provided the funding. H-SP and H-JK wrote the paper. All authors contributed to the article and approved the submitted version.

## Funding

This study was supported by the Basic Science Research Program through the National Research Foundation of Korea (NRF) funded by the Ministry of Science, ICT and future Planning (2017R1A5A2015385 and 2020R1A2C1008826).

## Conflict of Interest

The authors declare that the research was conducted in the absence of any commercial or financial relationships that could be construed as a potential conflict of interest.

## Supplementary Material

Supplementary Figure 1 Confirmation of endotoxin decontamination of purified Rv2145c. **(A)** BMDMs were incubated with LPS (100 ng/ml), Rv2145c (10 μg/ml), boiled LPS, or boiled Rv2145c. **(B)** BMDMs were incubated with LPS or Rv2145c in the presence and absence of proteinase K (PK). **(C)** BMDMs were incubated with LPS or Rv2145c in the presence and absence of PMB. After 24 h, IL-10, TNF-α, and IL-12p70 production in the culture supernatants was analyzed by ELISA. All data are expressed as the mean ± SD (*n* = 3). **p* < 0.05, ***p* < 0.01, and ****p* < 0.001 for treatment compared with the difference between treatment data. *n.s.*, no significant difference.

Supplementary Figure 2 Rv2145c-mediated Mtb growth enhancement is not observed in BMDMs from TLR4^–/–^ mice. BMDMs derived from WT, TLR2^–/–^ and TLR4^–/–^ mice were infected with Mtb at an MOI of 1 for 4 h and incubated with or without 10 μg/ml Rv2145c for 72 h. Intracellular bacterial growth was analyzed by CFU assay at 0 and 72 h. Similar results were obtained in three independent experiments. ***p* < 0.01 compared with infection controls (MC). Treatments with no significant effect are indicated as *n.s.*

Supplementary Figure 3 Anti-Rv2145c antibodies suppress Mtb growth in macrophages. BMDMs were pre-treated with antiserum against Rv2145c or PBS for 1 h, infected with Mtb at an MOI of 1 for 4 h, and incubated in the presence and absence of antiserum for the indicated times. CFU assays were conducted at the indicated times. **p* < 0.05 for treatment compared with medium controls (MC). *n.s.*, no significant difference.

Supplementary Figure 4 Confirmation of *Mycobacterium smegmatis* expressing Rv2145c and a vector control strain. **(A)** Ms_vector and Ms_Rv2145c were grown at 37°C in 7H9 liquid medium to an OD_600_ of 0.6–1.0. The bacterial lysates were subjected to Western blot analysis to detect His-tagged Rv2145c using mouse anti-His antibody. **(B)** Growth of Ms_vector and Ms_Rv2145c at 37°C in 7H9 liquid medium was monitored by determining the OD_600_ at intervals of 8 h.

Supplementary Figure 5 Confirmation of siRNA transfection for STAT3 gene silencing. BMDMs transfected with STAT3 siRNA (siSTAT3) or nonspecific siRNA as a control (siCON) were infected with Mtb at an MOI of 1 for 4 and incubated with or without 10 μg/ml Rv2145c for 48 h. The protein expression of phospho-STAT3, STAT3 and β-actin in the BMDMs was analyzed by Western blot analysis. The image is representative of three experiments showing similar results.

Supplementary Figure 6 The Rv2145c C-terminal region is active in modulating Mtb growth. **(A)** The full-length Rv2145c protein was separated into the N-terminal part (D1, aa 1 to 90) and C-terminal part (D2, aa 91 to 260). Each truncated protein was expressed in *E. coli*, purified, and analyzed by SDS-PAGE. **(B)** BMDMs were stimulated with full-length Rv2145c (10 μg/ml), Rv2145c D1 (1, 5, and 10 μg/ml) or Rv2145c D2 (1, 5, and 10 μg/ml) for 24 h, and the levels of IL-10, IL-6, and TNF-α in the culture supernatants were determined by ELISA. **(C)** Confirmation of endotoxin decontamination of the purified recombinant proteins. BMDMs were incubated with LPS or the proteins in the presence and absence of PMB. After 24 h, IL-10, TNF-α, and IL-12p70 production in the culture supernatants was analyzed by ELISA. **(D)** BMDMs were infected with Mtb at an MOI of 1 for 4 h and then further treated with gentamicin to kill extracellular bacteria for 2 h and incubated with or without 10 μg/ml Rv2145c, Rv2145c D1 or Rv2145c D2 for 72 h. Intracellular bacterial growth was determined by plating the cell lysates on 7H10 agar for 0 to 72 h. The mean ± SD is shown for three independent experiments. **p* < 0.05, ***p* < 0.01 and ****p* < 0.001 for treatment compared with medium controls (MC) or for the difference between treatment data. *n.s.*, no significant difference.

Supplementary Table 1 Sequences of primers, restriction sites, and vectors used for the recombinant proteins.

Supplementary Table 2 Information on the antibodies used this study.
