# Supplementary figures and images for: Mycobacterium tuberculosis Rv2145c Promotes Intracellular Survival by STAT3 and IL-10 Receptor Signaling
Source: Front Immunol. 2021 May 4;12:666293. doi: 10.3389/fimmu.2021.666293 (PMC8129509; doi:10.3389/fimmu.2021.666293)

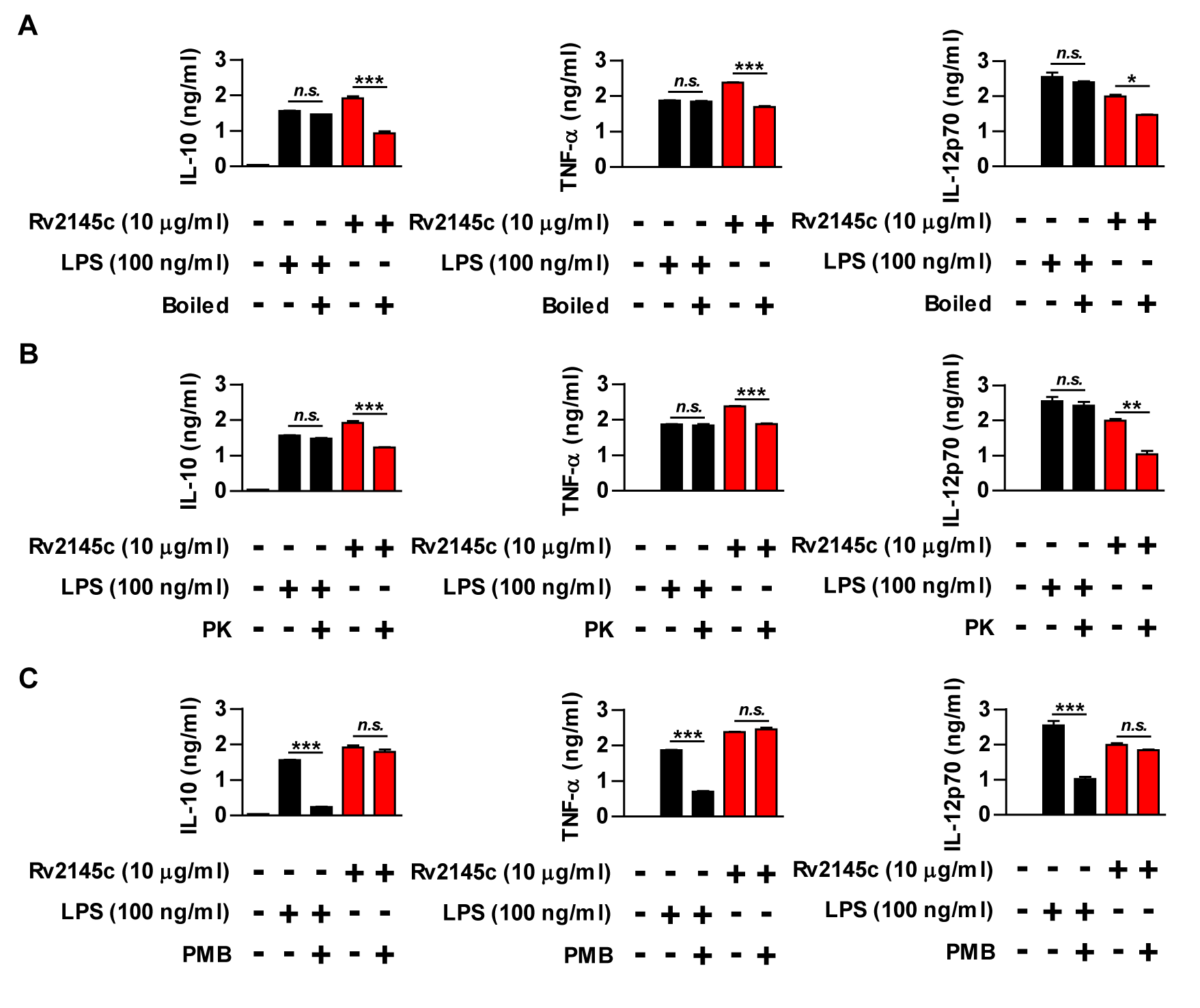

Supplement: Supplementary Figure 1 — Confirmation of endotoxin decontamination of purified Rv2145c. (A) BMDMs were incubated with LPS (100 ng/ml), Rv2145c (10 μg/ml), boiled LPS, or boiled Rv2145c. (B) BMDMs were incubated with LPS or Rv2145c in the presence and absence of proteinase K (PK). (C) BMDMs were incubated with LPS or Rv2145c in the presence and absence of PMB. After 24 h, IL-10, TNF-α, and IL-12p70 production in the culture supernatants was analyzed by ELISA. All data are expressed as the mean ± SD (n = 3). *p < 0.05, **p < 0.01, and ***p < 0.001 for treatment compared with the difference between treatment data. n.s., no significant difference. [file Image_1.tif]

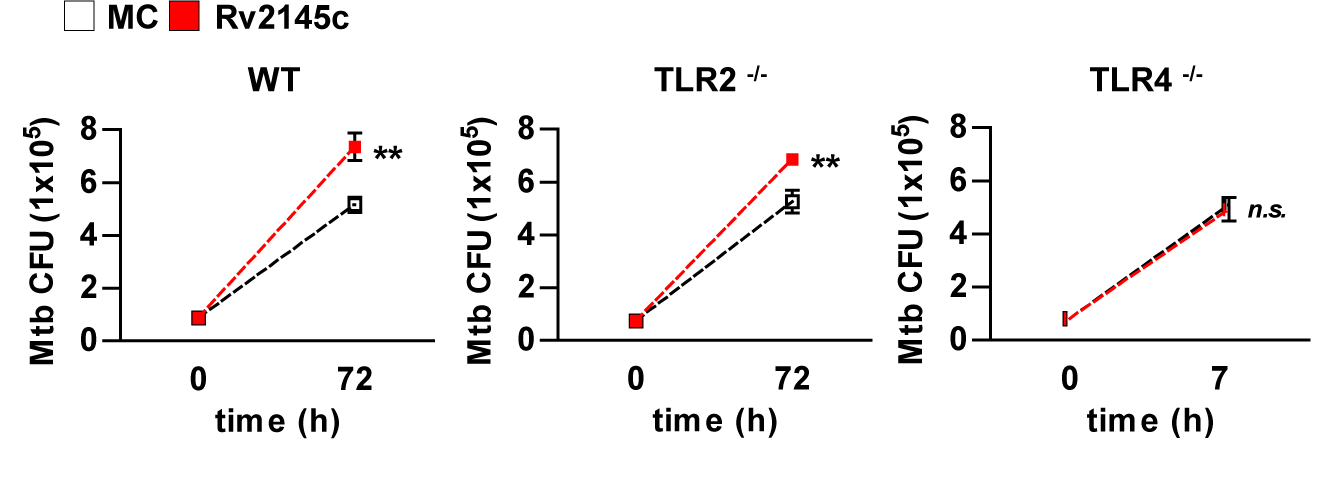

Supplement: Supplementary Figure 2 — Rv2145c-mediated Mtb growth enhancement is not observed in BMDMs from TLR4–/– mice. BMDMs derived from WT, TLR2–/– and TLR4–/– mice were infected with Mtb at an MOI of 1 for 4 h and incubated with or without 10 μg/ml Rv2145c for 72 h. Intracellular bacterial growth was analyzed by CFU assay at 0 and 72 h. Similar results were obtained in three independent experiments. **p < 0.01 compared with infection controls (MC). Treatments with no significant effect are indicated as n.s. [file Image_2.tif]

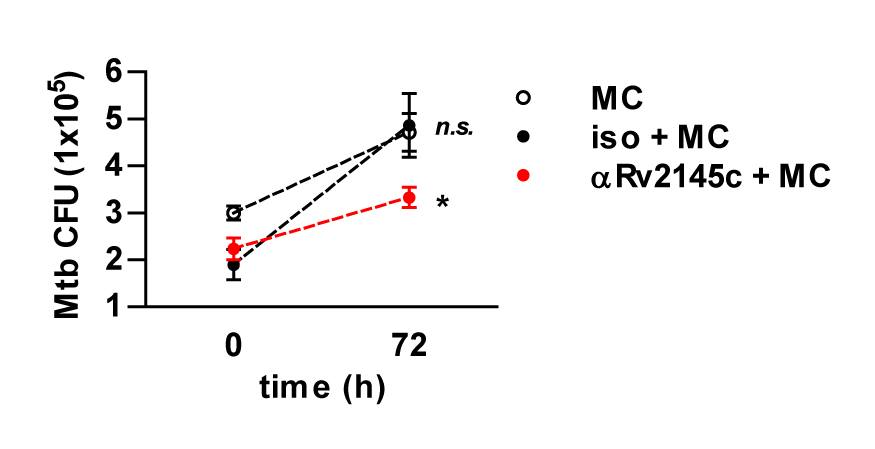

Supplement: Supplementary Figure 3 — Anti-Rv2145c antibodies suppress Mtb growth in macrophages. BMDMs were pre-treated with antiserum against Rv2145c or PBS for 1 h, infected with Mtb at an MOI of 1 for 4 h, and incubated in the presence and absence of antiserum for the indicated times. CFU assays were conducted at the indicated times. *p < 0.05 for treatment compared with medium controls (MC). n.s., no significant difference. [file Image_3.tif]

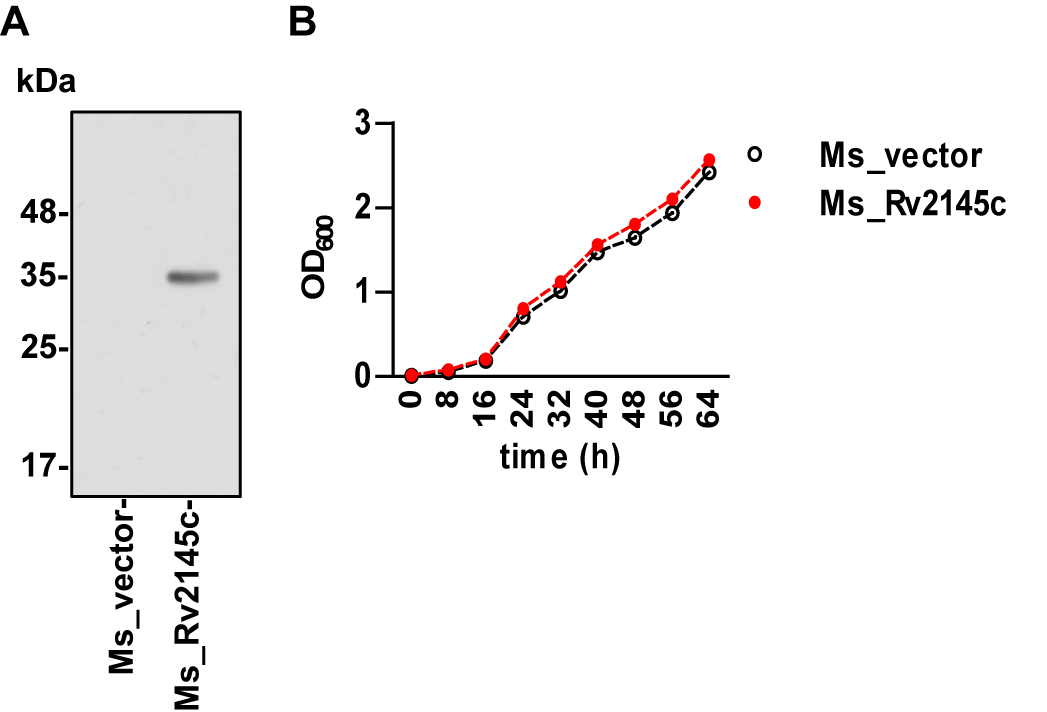

Supplement: Supplementary Figure 4 — Confirmation of Mycobacterium smegmatis expressing Rv2145c and a vector control strain. (A) Ms_vector and Ms_Rv2145c were grown at 37°C in 7H9 liquid medium to an OD600 of 0.6–1.0. The bacterial lysates were subjected to Western blot analysis to detect His-tagged Rv2145c using mouse anti-His antibody. (B) Growth of Ms_vector and Ms_Rv2145c at 37°C in 7H9 liquid medium was monitored by determining the OD600 at intervals of 8 h. [file Image_4.tif]

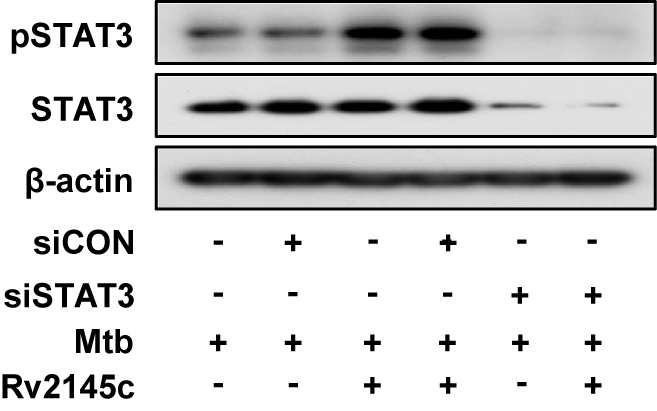

Supplement: Supplementary Figure 5 — Confirmation of siRNA transfection for STAT3 gene silencing. BMDMs transfected with STAT3 siRNA (siSTAT3) or nonspecific siRNA as a control (siCON) were infected with Mtb at an MOI of 1 for 4 and incubated with or without 10 μg/ml Rv2145c for 48 h. The protein expression of phospho-STAT3, STAT3 and β-actin in the BMDMs was analyzed by Western blot analysis. The image is representative of three experiments showing similar results. [file Image_5.tif]

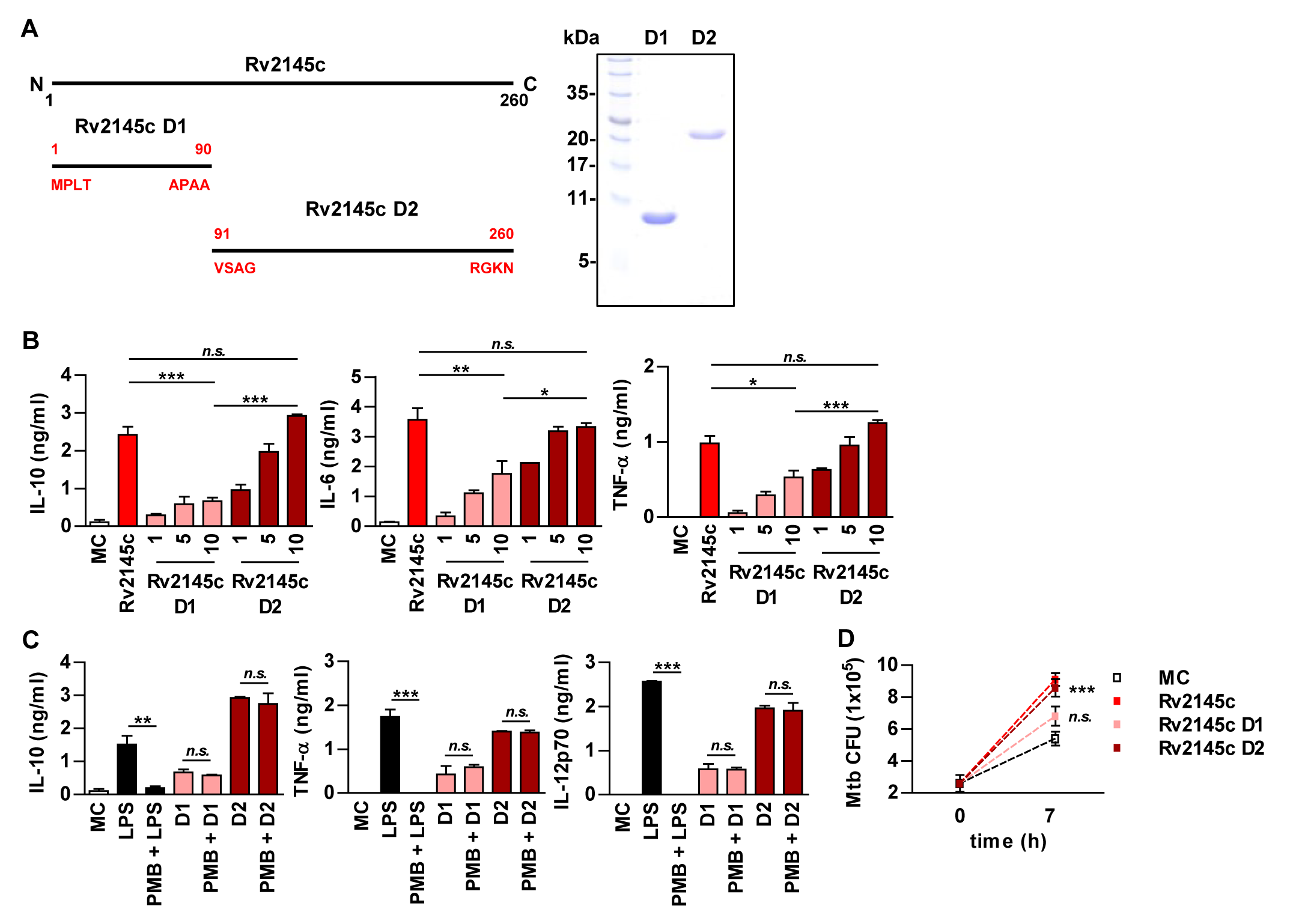

Supplement: Supplementary Figure 6 — The Rv2145c C-terminal region is active in modulating Mtb growth. (A) The full-length Rv2145c protein was separated into the N-terminal part (D1, aa 1 to 90) and C-terminal part (D2, aa 91 to 260). Each truncated protein was expressed in E. coli, purified, and analyzed by SDS-PAGE. (B) BMDMs were stimulated with full-length Rv2145c (10 μg/ml), Rv2145c D1 (1, 5, and 10 μg/ml) or Rv2145c D2 (1, 5, and 10 μg/ml) for 24 h, and the levels of IL-10, IL-6, and TNF-α in the culture supernatants were determined by ELISA. (C) Confirmation of endotoxin decontamination of the purified recombinant proteins. BMDMs were incubated with LPS or the proteins in the presence and absence of PMB. After 24 h, IL-10, TNF-α, and IL-12p70 production in the culture supernatants was analyzed by ELISA. (D) BMDMs were infected with Mtb at an MOI of 1 for 4 h and then further treated with gentamicin to kill extracellular bacteria for 2 h and incubated with or without 10 μg/ml Rv2145c, Rv2145c D1 or Rv2145c D2 for 72 h. Intracellular bacterial growth was determined by plating the cell lysates on 7H10 agar for 0 to 72 h. The mean ± SD is shown for three independent experiments. *p < 0.05, **p < 0.01 and ***p < 0.001 for treatment compared with medium controls (MC) or for the difference between treatment data. n.s., no significant difference. [file Image_6.tif]
