# Supplementary material for: Mycobacterium tuberculosis Rv2145c Promotes Intracellular Survival by STAT3 and IL-10 Receptor Signaling
Source: Front Immunol. 2021 May 4;12:666293. doi: 10.3389/fimmu.2021.666293 (PMC8129509; doi:10.3389/fimmu.2021.666293)
Supplement: Supplementary Table 1 — Sequences of primers, restriction sites, and vectors used for the recombinant proteins. [file Table_1.docx]

Supplementary Material

| **primer** | **sequence** | **restriction**  **enzyme sites** | **cloning**  **vector** |
| --- | --- | --- | --- |
| Rv2145c- Forward | 5’-CATATGCCGCTTACACCTGCCGACGTC-3’ | *Nde*I | pET22b (+) |
| Rv2145c- Reverse | 5’-AAGCTTGTTTTTGCCCCGGTTGAATTG -3’ | *Hin*dIII |  |
| Ag85B- Forward | 5’-GAATTCGATGACAGACGTGAGCCGAAAG-3’ | *Eco*RI | pET22b (+) |
| Ag85B- Reverse | 5’-AAGCTTGCCGGCGCCTAACGAACTCTG-3’ | *Hin*dIII |  |
| Rv2145c D1- Forward | 5’-CATATGCCGCTTACACCTGCCGACGTC-3’ | *Nde*I | pET22b (+) |
| Rv2145c D1- Reverse | 5’-AAGCTTCGCCGCCGGCGCCGGCTTGCC-3’ | *Hin*dIII |  |
| Rv2145c D2- Forward | 5’-CATATGGTCTCGGCGGGGATGAACGAG-3’ | *Nde*I | pET22b (+) |
| Rv2145c D2- Reverse | 5’-AAGCTTGTTTTTGCCCCGGTTGAATTG -3’ | *Hin*dIII |  |

**Supplementary Table 1.** **Sequences of primers, restriction sites, and vectors used for the recombinant proteins.**
