# Supplementary material for: Mycobacterium tuberculosis Rv2145c Promotes Intracellular Survival by STAT3 and IL-10 Receptor Signaling
Source: Front Immunol. 2021 May 4;12:666293. doi: 10.3389/fimmu.2021.666293 (PMC8129509; doi:10.3389/fimmu.2021.666293)
Supplement: Supplementary Table 2 — Information on the antibodies used this study. [file Table_2.docx]

Supplementary Material

| **antibody name** | **company** | **catalog number** | **experiment** |
| --- | --- | --- | --- |
| HIS (HIS.H8) | Abcam | ab18184 | WB |
| groEL | Abcam | ab90522 | WB |
| mouse iNOs (54/iNOS) | BD Biosciences | 610431 | WB |
| CD126 (D7715A7)-PE | BD bioscience | 554462 | FACS |
| CD210 (1B1.3a)-PE | BD bioscience | 559914 | FACS |
| IgG isotype control (HRPN) | Bio X Cell | BE0088 | Blocking |
| IgG2b isotype control (LTF-2) | Bio X Cell | BE0090 | Blocking |
| mouse IL-6R (15A7) | Bio X Cell | BE0047 | Blocking |
| mouse IL-10R (CD210, 1B1.3A) | Bio X Cell | BE0047 | Blocking |
| mouse IgG-HRP | Calbiochem | 401215 | WB |
| rabbit IgG-HRP | Calbiochem | 401353 | WB |
| pERK1/2 (T202/Y204) | Cell Signaling Technology | 9101 | WB |
| pp38 (T180/Y182) | Cell Signaling Technology | 9211 | WB |
| pJNK (T183/Y185) | Cell Signaling Technology | 4671 | WB |
| pIκB-α (S32, 14D4) | Cell Signaling Technology | 2859 | WB |
| IκB-α (44D4) | Cell Signaling Technology | 4812 | WB |
| pSTAT3 (Tyr705, D3A7) | Cell Signaling Technology | 9145 | WB, Confocal |
| STAT3 (124H6) | Cell Signaling Technology | 9139 | WB |
| pSTAT1 (Tyr701, 58D6) | Cell Signaling Technology | 9167 | WB |
| STAT1 | Cell Signaling Technology | 9172 | WB |
| NF-κB p65 (D14E12) | Cell Signaling Technology | 8242 | Confocal |
| β-actin (13E5) | Cell Signaling Technology | 4970 | WB |
| F4/80 (BM8)-APC | eBioscience | 17-4801-82 | FACS |
| CD80 (16-10A1)-PE | eBioscience | 12-0801-83 | FACS |
| CD86 (GL1)-PE | eBioscience | 12-0862-83 | FACS |
| MHC class I (34-1-2S)-PE | eBioscience | 12-5998-83 | FACS |
| MHC class II (I-A/I-E, M5/114.15.2)-PE | eBioscience | 12-5321-83 | FACS |
| mouse TNF-α | eBioscience | 88-7324-77 | ELISA |
| mouse IL-6 | eBioscience | 88-7064-77 | ELISA |
| mouse IL-10 | eBioscience | 88-7105-77 | ELISA |
| mouse IL-12p70 | eBioscience | 88-7121-77 | ELISA |
| Texas Red®-X phalloidin | Molecular Probes | T7471 | Confocal |
| TLR2 (H-175) | Santa Cruz Biotechnology | sc-10739 | WB |
| TLR4 (25) | Santa Cruz Biotechnology | sc-293072 | WB |
| mouse IgG- Alexa Fluor 488 | Thermo Fisher Scientific | A11001 | Confocal |
| rabbit IgG- Alexa Fluor 488 | Thermo Fisher Scientific | A11008 | Confocal |
| IgG isotype control (EBRG1) | Thermo Fisher Scientific | 16-4301-81 | Neutralization |
| mouse IL-6 (MP5-20F3) | Thermo Fisher Scientific | 16-7061-81 | Neutralization |
| mouse IL-10 (JE35-2A5) | Thermo Fisher Scientific | 16-7102-85 | Neutralization |

Abcam (Abcam, Cambridge, UK), Bio X Cell (Lebanon, Pennsylvania, USA), Calbiochem (San Diego, CA, USA), Cell Signaling Technology (Danvers, MA, USA), eBioscience (San Diego, CA, USA), Santa Cruz Biotechnology (Paso Robles, CA, USA), Thermo Fisher Scientific (Waltham, MA, USA).

**Supplementary Table 2. Information on the antibodies used this study.**
